# Upregulation of Defensins in Burn Sheep Small Intestine

**Published:** 2009-12-26

**Authors:** Brian J. Poindexter, Gordon L. Klein, Stephen M. Milner, Roger J. Bick

**Affiliations:** ^a^ Department of Pathology and Laboratory Medicine, University of Texas Medical School, Houston; ^b^ Department of Pediatrics, Section of Gastroenterology, University of Kentucky, Lexington; ^c^ Johns Hopkins University Burn Center, Baltimore, Md; and Johns Hopkins University Wound Healing Center, Baltimore, Md.

## Abstract

**Objective:** The aim of this study was to visualize and localize the sheep antimicrobials, β-defensins 1, 2, and 3, (SBD-1, SBD-2, SBD-3), sheep neutrophil defensin alpha (SNP-1), and the cathelicidin LL-37 in sheep small intestine after burn injury, our hypothesis being that these compounds would be upregulated in an effort to overcome a compromised endothelial lining. Response to burn injury includes the release of proinflammatory cytokines and systemic immune suppression that, if untreated, can progress to multiple organ failure and death, so protective mechanisms have to be initiated and implemented. **Methods:** Tissue sections were probed with antibodies to the antimicrobials and then visualized with fluorescently labeled secondary antibodies and subjected to fluorescence deconvolution microscopy and image reconstruction. **Results:** In both the sham and burn samples, all the aforementioned antimicrobials were seen in each of the layers of small intestine, the highest concentration being localized to the epithelium. SBD-2, SBD-3, and SNP-1 were upregulated in both enterocytes and Paneth cells, while SNP-1 and LL-37 showed increases in both the inner circular and outer longitudinal muscle layers of the muscularis externa following burn injury. Each of the defensins, except SBD-1, was also seen in between the muscle layers of the externa and while burn caused slight increases of SBD-2, SBD-3, and SNP-1 in this location, LL-37 content was significantly decreased. **Conclusion:** That while each of these human antimicrobials is present in multiple layers of sheep small intestine, SBD-2, SBD-3, SNP-1, and LL-37 are upregulated in the specific layers of the small intestine.

Postburn infection and associated high levels of circulating proinflammatory cytokines and other mediators induce systemic inflammatory response syndrome, immunosuppression, and sepsis, leading to the possibility of multiple organ failure and death.^1^,^2^ Previous studies from our laboratory have demonstrated the induction of the antimicrobial peptide (AMP) human β-defensin-2 (HBD-2) by TNF-α in cultured keratinocytes and IL-1β-induced cell hypertrophy.^3^ In addition, we reported significantly reduced levels of HBD-2 in burn skin epidermis, and its absence from burn blister fluids. HBD-2 is also poorly expressed in lung fluid obtained after inhalation injury.^4^^-^7 However, in our samples of burn skin, we were able to localize defensins to specific cells and structures in the deeper dermis, and even hypodermis, of both normal and burn skin, suggesting specific roles for the peptides and the possibility of upregulation of antimicrobials even when the epidermis has been destroyed.^8^,^9^

AMPs are important components of the innate immune system, playing a major role in body defence as regulators of microbial density in the small intestine and in the protection of nearby stem cells.^10^^-^13 Defensins are small (29–45 amino acids) cationic peptides that have been divided into 2 main families, α- and β-defensins, on the basis of the disulphide bond pairing pattern.^13^ The defensins are synthesized as precursor polypeptides and are then posttranslationally processed into mature active peptides.^10^^-^13

In humans, 4 neutrophil defensins (HNP-1, -2, -3, -4) were first identified, followed by 2 enteric α-defensins (HD-5, -6), the expression of which is normally restricted to Paneth cells, an enteric epithelial cell lineage specific to mammalian small intestinal crypts.^13^^-^15 Enteric α-defensins have been well characterized and are termed “cryptdins” (crypt defensins) and are major constituents of neutrophil azurophilic granules and are also released as secretory granule components of Paneth cells.^14^^-^16 In contrast, human β-defensin 1 (HBD-1) and other members of the β-defensin family appear to be expressed by most epithelial cells of the small and large intestine, HBD-1 being expressed constitutively, while HBD-2 synthesis is induced by activation of the transcriptional factor NFκB.^12^,^13^ HBD-2 is perhaps the most relevant β-defensin in the gastrointestinal tract, where it is expressed only when infection or inflammation is present, its induction being mediated by proinflammatory cytokines through NF-κB and AP-1-dependent pathways,^12^,^13^ while HBD-3 and 4 are also inducible and are expressed by the small intestinal, particularly in the crypts, and colonic epithelial cells.^13^ Little is known about the distribution, regulation, and production of cathelicidin LL-37 within the intestinal mucosa, although some studies noted the presence of mRNA and protein expression in the human colon and LL-37 mRNA in the epithelium of the small intestine.^13^,^17^

We employed fluorescence deconvolution microscopy and image reconstruction to determine which antimicrobials subject to increases and/or decreases after burn injury, while also determining in which specific cell type(s) antimicrobial synthesis occurs, in order to further understand the protective responses to the systemic effects of burn.

## METHODS

All chemicals were purchased from Sigma Chemical Corp (St Louis, Mo) except where stated and are of the highest grade available.

## TISSUE PREPARATION

The sheep model of burn is well-established and has been used to study the cardiopulmonary effects of thermal burns for more than 20 years.^16^,^17^ This model has been reviewed and approved by the institutional Animal Care and Use Committee, University of Texas Medical Branch, and samples were generously supplied by Dr D. Traber (UTMB-Galveston, Department of Anesthesiology). Cardiopulmonary effects of burn injury in sheep are similar to those in humans and there are also similarities in the effects of burn on the bones of sheep when compared to humans.^16^^-^18 Ewes of the Merino breed obtained from The University of Texas Medical Branch Animal Resource Center were subjected to a controlled, full-thickness flame burn on the back and sides covering 40% of the total body surface area while under halothane anesthesia. Sensory nerve endings are destroyed during this process, and no pain is felt by the animal.^16^^-^19 Sham animals also received halothane. The sheep were then given intravenous fluid resuscitation according to the Parkland formula and subsequently kept in a standing position in metabolic cages and fed standard Purina sheep chow (Ralston Purina Company, St Louis, Mo), with free access to food and water.^16^,^17^ At the end of the study period, the sheep were sacrificed, and tissue samples were removed immediately and transferred to UT-Houston on ice or embedded in sucrose-based OCT compound (Tissue-Tek, Torrance, Calif) and frozen on dry ice.

Processing of samples and methodology for fluorescence deconvolution microscopy has previously been presented and discussed in detail.^7^,^8^,^20^ Briefly, frozen sections were cut at a thickness of 12 ± 3 μm with a Microm HM 505 E cryotome (Microm Laboratories, Walldorf, Germany) and placed on 18-mm glass cover slips (Fisher, Pittsburgh, Pa), which had been acid cleaned and coated with poly-L-lysine. Sections were rinsed with cold phosphate-buffered saline, fixed in 3.7% paraformaldehyde (Tousimis Research, Rockville, Md) at room temperature (RT), and rinsed 5 times with RT phosphate buffered saline. Cover slips were inverted and floated on 10% goat serum for 1 hour at 37°C to reduce nonspecific antibody binding, then antibodies against human defensins HBD-1, HBD-2, HNP-1 (Alpha Diagnostic, San Antonio, Tex), HBD-3 (Novus Biologicals, Littleton, Colo), LL-37 (Hycult Biotechnology b.v., Uden, The Netherlands), and smooth muscle actin (Sigma, St Louis, Mo) were diluted 1:100 in 10% goat serum and incubated with the sections for 45 minutes at 37°C. After rinsing the coverslips in 0.05% Tween-20, fluorescently tagged secondary antibodies (Molecular Probes, Eugene, Ore) were added and the sections were incubated for 30 minutes at 37°C and, finally, F-actin and the nuclei were simultaneously stained with phallicidin and DAPI (Molecular Probes, Eugene, Ore), respectively, for 15 minutes at RT. Cover slips were mounted onto glass slides with Elvanol (DuPont, Wilmington, Del) as the mounting media and attached with nail polish.

Specificities of binding of fluorescent antibodies to defensins and cathelicidin were determined by the inclusion of cold protein in labeling experiments, prior to the addition of antibody. Secondary (fluorescent) antibody labeling following this “cold protein wash” was <8% of total in all cases.

## RECONSTRUCTIVE MICROSCOPY (DECONVOLUTION)

Specimens were scanned with an Applied Precision DeltaVision (Issaquah, Wash) system fitted with an Olympus IX 70 inverted microscope employing a 100-W mercury arc lamp for illumination (Olympus America, Melville, NY) and excitation/emission filter sets (Chroma Technology Corp, Brattleboro, Vt) specific for each of the fluorescent antibodies. The filter set combination for DAPI (nucleus) was a 340-nm excitation filter with a bandpass of 20 nm and an emission filter of 390 nm with a bandpass of 20 nm. Phallicidin (F-actin) fluorescence is acquired with an excitation filter of 488 nm (bandpass 10 nm) and an emission filter of 520 nm (bandpass 25 nm). Defensin antibodies were visualized with an excitation filter of 555 nm (bandpass of 28 nm) and an emission filter of 617 nm (bandpass of 73 nm) and smooth muscle actin was acquired with an excitation filter of 640 nm (bandpass 20 nm) and emission filter of 685 (bandpass of 40 nm). Image scans for each probe were acquired in a series at a step size of 0.35 μm with a Sony Interline CCD camera. At least 30 sections are scanned per sample for each probe (ie, 120 total images for the 4 probes used). Specimen magnification is 200× unless otherwise noted.

Deconvolution and image analysis were performed by transferring the data sets to a Linux/RedHat workstation employing SoftWoRx software (Applied Precision), which uses an algorithm experimentally produced on the system from the convolution of a point spread function (PSF) to differentiate and reduce extraneous light or scattered light captured by the camera. A PSF describes the imaging and resolution characteristics of light collected by the optics of the microscope and is derived by scanning a 0.1-μm fluorescent bead (Molecular Probes, Eugene, Ore) 4 μm above and 4 μm below the plane of focus. The resulting PSF is Fourier transformed into an optical transfer function that manipulates the data to produce images with a higher signal-to-noise resolution of the probe emission patterns. All data sets are subjected to 10 deconvolution iterations and then used for image analysis, image reconstructions, volume rendering, and modeling. Subtraction of background fluorescence and change of intensity gain were optimally set for each emission.

An image projection is produced by stacking each of the individual *z*-sections of all 3 fluorescent probes into one image, resulting in a 3-dimensional (3D) end product and an overlay of all colors. Volume rendering uses the stack of *z*-sections and rotates them about the X or Y plane. Each of the volume rotation movies is produced with a 6° view angle. A 3D computer-generated virtual model of the fluorescence emission patterns was produced to view the relative positions of each emission pattern and elucidate localization of the defensins to specific cells types. Briefly, positive and background intensities are measured and thresholds set to produce polygons on the basis of the increased signal-to-noise ratio of the positive signal intensities. The software uses these polygons to generate 3D objects and a virtual model that represents the emission patterns of each fluorescent probe. The 3D model is fully interactive and can be rotated in any plane for viewing at any angle or perspective.

## RESULTS

**Figure 1:**
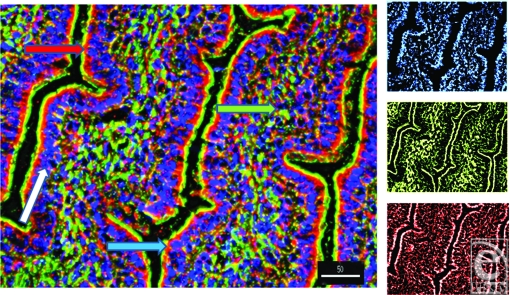
Longitudinal image of villi to demonstrate the localization of the antimicrobial SBD-3 (red) in the epithelial cells of the small intestine. Blue (DAPI) indicates cell nuclei and green (Phallicidin) shows F-actin. Note the green microvillous, brush border on the apical side of the cells (magnification ×200; deconvolution via 5 iterations). Red arrow shows SBD-3 in the apex of the intestinal epithelium, the green arrow points to the lacteal, the blue arrow indicates the microvilli, and the white arrow points along the layer of intestinal epithelial cells. Scale bar = 50 microns. The smaller panels on the left are split channels images to show nuclei (top), F-actin (middle panel), and SBD-3 (bottom panel).

**Figure 2:**
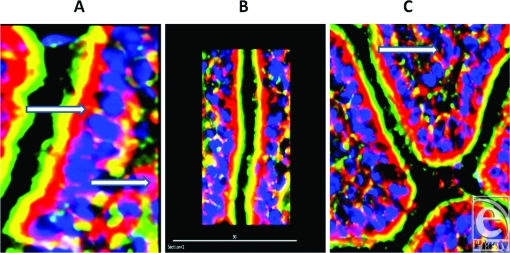
Longitudinal image of villi to demonstrate the localization of the antimicrobial SBD-3 (red) in the epithelial cells of the small intestine. Blue (DAPI) indicates cell nuclei and green (Phallicidin) shows F-actin. Note the green microvillous, brush border on the apical side of the cells (magnification ×200; deconvolution via 5 iterations). Panel A is magnification ×1200, Panel B is magnification ×500, and Panel C is magnification ×500. White arrows indicate SBD-3 in the epithelium and subepithelial capillaries in the panel A and are associated with the lacteal and surrounding capillaries in panel C. Scale bar in panel B = 90 microns.

**Figure 3:**
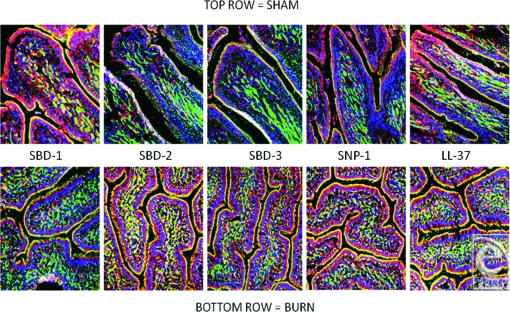
Comparison of all the targeted peptides with sham samples on the top row, burn samples on the bottom. Magnification ×200. Red = peptide as indicated (orange/yellow = colocalized red and green probes), blue = nuclei, green = F-actin.

Alpha and β-defensins were located in each of the layers of the burn sheep small intestine (mucosa, submucosa, and externa) in both sham and burn samples, with the highest concentrations in the luminal epithelium. Luminal enterocytes showed intense staining for all defensins and exhibited consistently high concentrations in the apical side of the cells (Fig 1; Video 1). Furthermore, most cells were densely packed with defensins, as evidenced by intense staining throughout the cytoplasm (Fig 2; Video 2). Figures 2a–2c show high magnification renditions of areas or the villus, demonstrating the defensins (red) within individual cells and colocalized (yellow) with basal aspects of the microvilli (green). When burn samples were compared to sham tissues, SBD-2, SBD-3, and SNP-1 content was increased, SBD-1 content was decreased, and there was little or no change in LL-37 content (Fig 3, antimicrobials shown in red).

**Figure:**
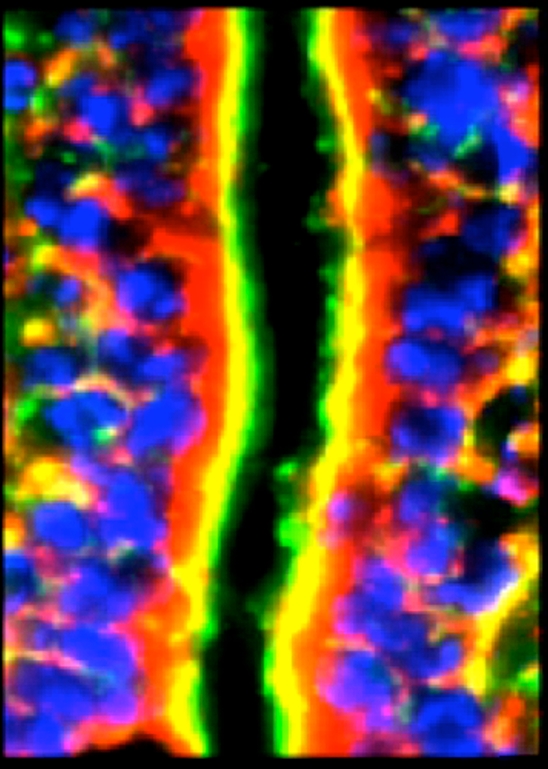
Video 1. “This video is in QuickTime format. If you do not have QuickTime you may download it here”.

**Figure:**
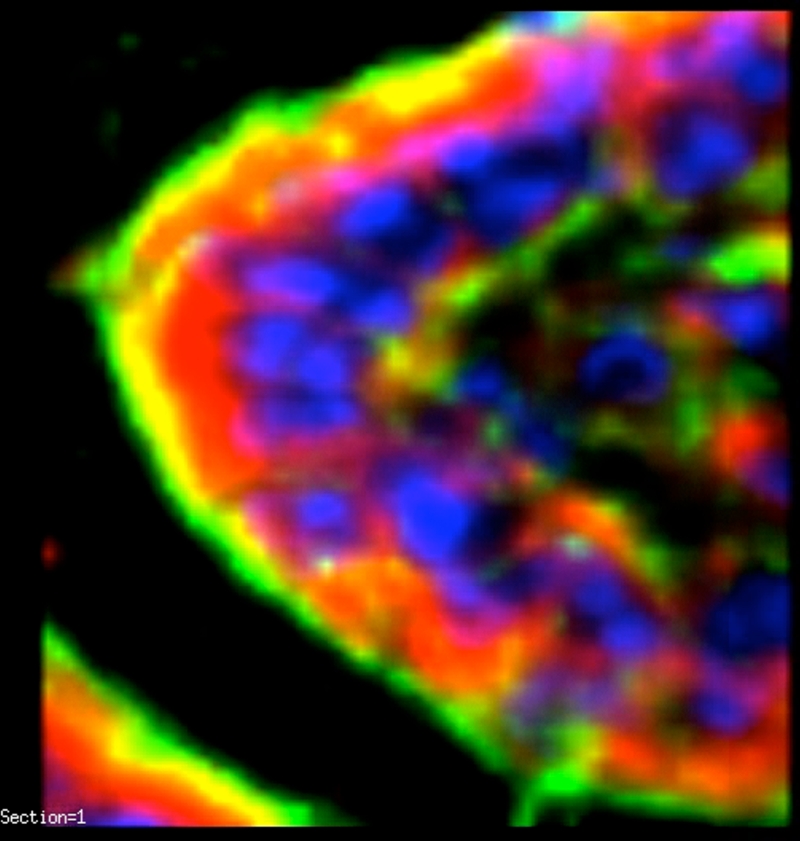
Video 2. “This video is in QuickTime format. If you do not have QuickTime you may download it here”.

**Figure 4:**
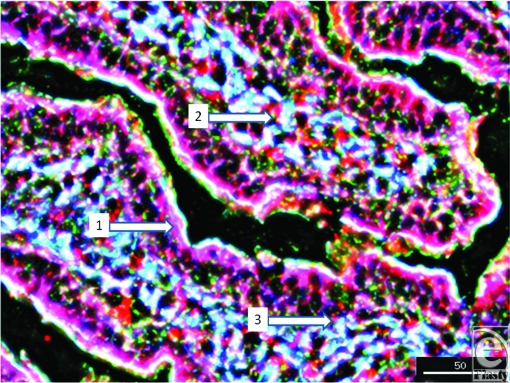
Image of a villus to show the smooth muscle actin of the vasculature (blue) located juxtapositioned to the lacteal. This particular image (magnification ×200) shows SBD-1 in red, F-actin is green, and smooth muscle actin is blue. Scale bar = 50 microns. White arrows indicate 1, intraepithelial SBD-1; 2, perilacteal capillary SBD-1; and 3, subepithelial capillary-associated SBD-1.

**Figure 5:**
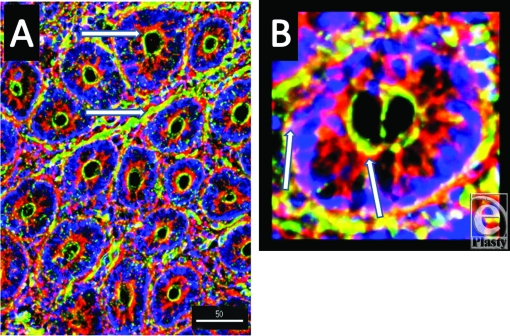
A low-power acquisition (magnification ×200) and a high-power (magnification ×500) outtake of cross sections of crypts to show the localization of SBD-3 to both the apical and basal aspects of the epithelial cells. Red = SBD-3; green = F-actin; and blue = nuclei. Scale bar = 50 microns in low-power image. White arrows indicate epithelial cells and capillaries in panel A and both the basal and apical aspects of cells in panel B.

To further localize the defensins (red), we probed for smooth muscle actin (blue) to show the microvasculature around the lacteal within the villi (Fig 4), where each of the four studied antimicrobials (red and white) was seen associated with the perilacteal capillaries (blue). Figure 5 and videos 3 and 4 show examples of the localization of peptides in cross sections of the intestinal crypts, as evidenced by the green brush border, just above the lamina propria and muscularis mucosa, the central space being the lumen of the gastrointestinal tract. The apical portion of the intestinal endothelial cells is seen as the red, inner circle around the central spaces, and once again demonstrates large amounts of peptide (SBD-3; panel 5a) and SPN-1 (high power) in the lining cells. As before, all of the defensins were present within the Paneth cells.

**Figure:**
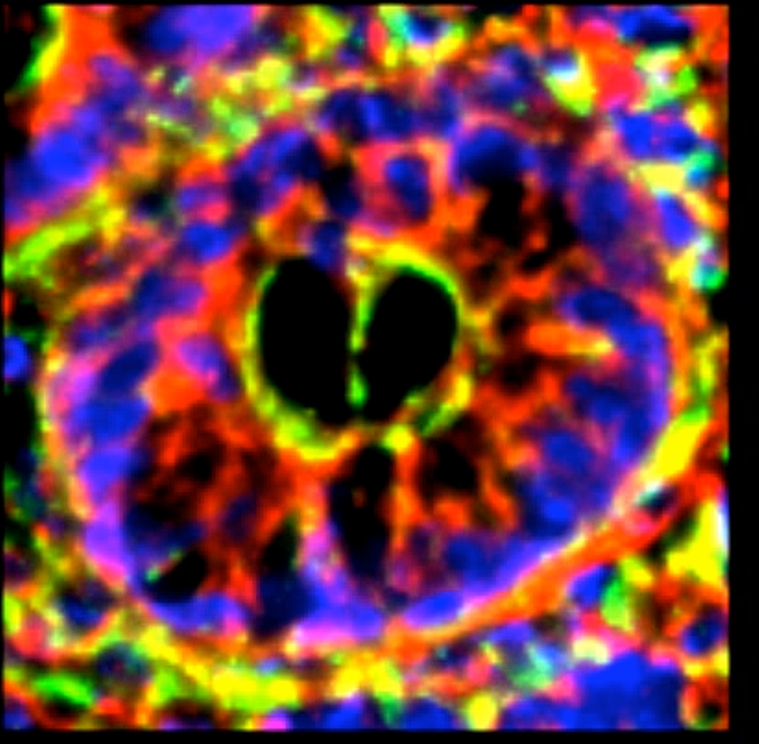
Video 3. “This video is in QuickTime format. If you do not have QuickTime you may download it here”.

**Figure:**
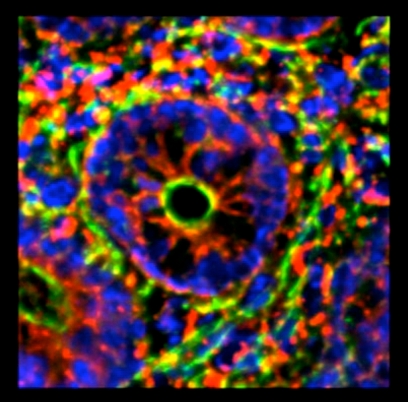
Video 4. “This video is in QuickTime format. If you do not have QuickTime you may download it here”.

**Figure 6:**
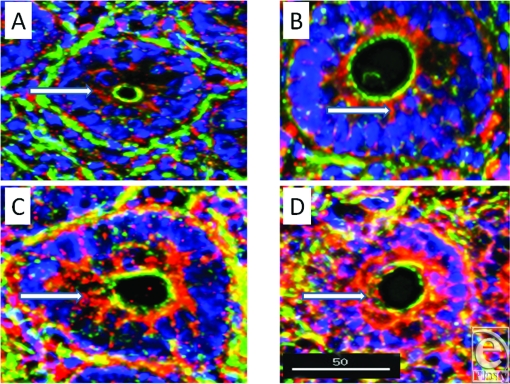
Comparison of cross sections of crypts from small intestine of sham and burn animals (magnification ×300). Note the dramatic increases in content of SBD-3 (panels A and C) and LL-37 (panels B and D) in the intestinal epithelial cells in these burn tissues (panels C and D), compared with sham-operated sections (panels A and B). Red = peptide; green = F-actin; and blue = nuclei. Scale bar = 50 microns.

A substantial increase in all defensins, except SBD-1, was observed following burn injury (Fig 6), with increases seen throughout the cytoplasm of the lining cells as shown in the lower panels of these cross-sectional images localizing SBD-3 and LL-37.

**Figure 7:**
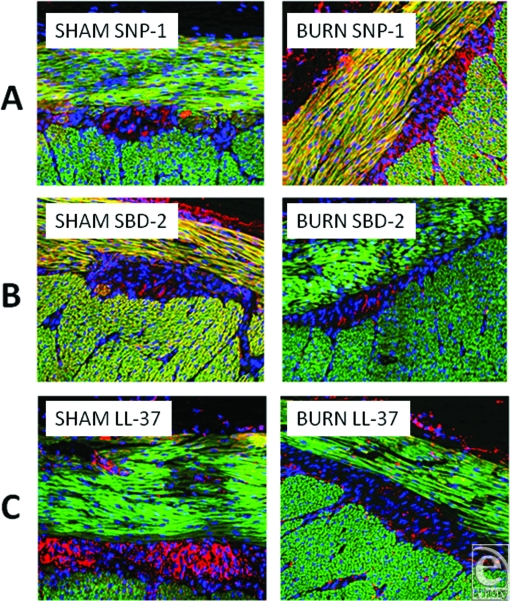
Three pairs of images, showing changes in content in the outer, externa, aspects of small intestine samples. Panel A shows 2 images to compare the dramatic increase of SNP-1 following burn injury, panel B shows the dramatic decrease in SBD-2, and panel C shows the dramatic decrease in LL-37 following burn injury in the region of the Auerbach's plexus. Magnification ×200; red = peptide, green = F-actin, and blue = nuclei.

To describe the location of defensins, images were constructed of the outer, muscularis externa, and adventitia (Fig 7). The upper two panels (a) show the dramatic increase in content of SNP-1 in the 2 layers of the externa (red/orange). However, the lower panels (b) reveal that SBD-2 content was dramatically decreased in the muscularis externa. Between the 2 outer muscle layers resides the ganglia-containing Auerbach's plexus. LL-37 was clearly seen to be present in this layer (7c) but was virtually absent following burn, although slight, inconsistent increases of SBD-2, SBD-3, and SNP-1 did occur (images not shown).

**Figure 8:**
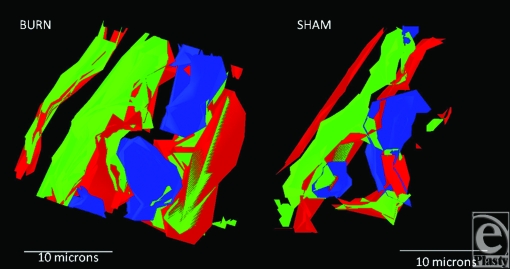
Generated 3D models of a burn versus sham tissue acquisition of SBD-3 in enterocytes of the villi. Note the perinuclear distribution of the peptide (red) as well as throughout the cytoplasm.

Finally, as an example of the models that are generated for precise location of component proteins and organelles, Figure 8 shows 2 models of Burn and Sham tissues, taken from the apical portion of the villi. Green is the F-actin cytoskeleton, blue are the enterocyte nuclei, and red is SBD-3. The amount of SBD-3 peptide in the burn was 8149 pixels versus 3332 pixels in the sham tissue, again identifying a large upregulation due to burn injury.

## DISCUSSION

Antimicrobial peptides play an important role in the natural defense armory of the body, and a loss of the capability to synthesize and release these compounds reduces our ability to combat infection and sepsis.^1^,^2^ Infection, coupled with the associated proinflammatory response and subsequent immunosuppression, contributes to multiple organ failure and death.^1^,^2^ Our earlier work^4^^-^7 demonstrated the loss of epidermal HBD2 after thermal injury, while showing that several defensins were located in deeper cells and specialized structures of the burned skin.^8^,^9^ As research investigating stress mechanisms was under way and burn sheep tissue was being used to localize ion control proteins, it was decided to use this opportunity to image samples of small intestine to localize natural antimicrobials before and after thermal injury in order to (i) see the cellular and location specificity of these peptides and (ii) ascertain how much of this natural defensive barrier against infection had been up- and/or downregulated, our hypothesis being that major changes would occur, following observations made in our studies with the ileal calcium sensing receptor, during which we noticed compromised tight junctions and changes in protein levels.^21^

The epithelium of the small intestine is composed of a single cell layer of columnar goblet cells and enterocytes, the latter secreting AMPs into a thin film on their luminal surface, forming an antimicrobial barrier that limits direct access by microbes to the epithelia and underlying lamina propria.^11^^-^13 In the intestine, 2 “systems” of AMP defense are recognized, that of the Paneth cells, which lie at the base of the small bowel crypts, and that of the enterocyte, which line the gut wall.^11^ In humans, defensins and cathelicidins are expressed by both circulating white blood cells and epithelial surfaces. Certain epithelial defensins are constitutively expressed (HBD-1), whereas others are induced after tissue injury or exposure to microbes (HBD-2, HBD-3, and HNP-1).^13^ Paneth cells are a major source of α-defensins and play a crucial role in innate immunity by limiting the number of microbes that colonize crypts and intestinal lumen, while also helping stabilize the composition of the endogenous flora.^15^,^16^ Paneth cells, with their armament of antimicrobial agents and their close proximity to noxious invaders, provide an effective protective mechanism against potential pathogenic insult. In this study, all the defensins were present in the cells of the crypts and, except for HBD-1, increased following burn injury. In addition, each of the defensins was located in the outer layers of the small intestine, the muscularis externa, submucosa, and serosa in both sham and burn sheep small intestine, suggesting that the microvasculature might well be intimately involved in protection, possibly as an ion source for the maintenance of muscle and neuromuscular activity.

Employing fluorescence deconvolution microscopy to produce 3-dimensional, volume-rendered images (computer-generated virtual models)^8^,^9^ allowed for the determination of which specific cell type(s) produced or included specific AMPs. The data showed that antimicrobial defense mechanisms were induced as a consequence of thermal injury, with SBD-1 being the only peptide to decrease in some areas. Levels of SBD-2, SBD-3, SNP-1, and LL-37 increased, while SNP-1 and LL-37 showed increases in the externa, but LL-37 decreased significantly in the outer layers and the myenteric plexus following burn injury.

This work gives us more insights into the upregulation of AMPs in the small intestine after thermal injury and demonstrates that fluorescence microscopy of biopsy samples makes this technique not only important in the research arena but also a valuable tool in clinical pathology. Perhaps targeted, selective upregulation of antimicrobial production by specific cells would be a beneficial adjunct in the treatment of wounds and for combating infection.

## References

[1] Sheridan RL, Tompkins RG, Herndon D (2001). Etiology and prevention of multiple organ failure. Total Burn Care.

[2] Schwacha MG (2003). Macrophages and post-burn immune dysfunction. Burns.

[3] Bick RJ, Poindexter BJ, Bhat S, Gulati S, Buja M, Milner SM (2004). Effects of cytokines and heat shock on defensin levels of cultured keratinocytes. Burns.

[4] Milner SM, Ortega MR (1999). Reduced antimicrobial peptide expression in human burn wounds. Burns.

[5] Ortega MR, Ganz T, Milner SM (2000). Human beta defensin in burn blister fluid. Burns.

[6] Milner SM, Cole A, Ortega MR (2003). Inducibility of HBD-2 in acute burns and chronic conditions of the lung. Burns.

[7] Milner SM, Bhat M, Buja M, Gulati S, Poindexter BJ, Bick RJ (2004). Expression of human beta defensin 2 in thermal injury. Burns.

[8] Poindexter BJ, Bhat S, Buja M, Bick RJ, Milner SM (2006). Localization of antimicrobial peptides in normal and burned skin. Burns.

[9] Poindexter BJ (2006). Immunofluorescence, deconvolution microscopy and image reconstruction of human defensins in normal and burned skin. Burns.

[10] Ganz T (1999). Defensins and host mechanisms. Science.

[11] Ganz T, Lehrer RI (1998). Antimicrobial peptides of vertebrates. Curr Opin Immunol.

[12] Selsted ME, Miller SI, Henschen AH, Ouellette AJ (1992). Enteric defensins: antibiotic peptide components of intestinal host defense. J Cell Biol.

[13] Cunliffe RN, Mahida YR (2004). Expression and regulation of antimicrobial peptides in the gastrointestinal tract. J Leukoc Biol.

[14] Jones DE, Bevins CL (1992). Paneth cells of the human small intestine express an antimicrobial peptide gene. J Biol Chem.

[15] Ouelette AJ (1999). Paneth cell antimicrobial peptides and the biology of the mucosal barrier. Am J Physiol.

[16] Oulette AJ (2006). Paneth cell alpha-defensin synthesis and function. Curr Top Microbiol Immunol.

[17] Hase KL, Leopard JD, Varki N, Kagnoff MF (2002). Cell differentiation is a key determinant of cathelicidin LL-37/human cationic antimicrobial protein 18 expression by human colon epithelium. Infect Immun.

[18] Klein GL, Kikuchi Y, Sherrard DJ, Simmons DJ, Biondo N, Traber DL (1996). Burn-associated bone disease in sheep: roles of immobilization and endogenous corticosteroids. J Burn Care Rehabil.

[19] Traber DL, Bohs CT, Carvajal HF, Linares HA, Miller TH, Larson DL (1979). Early cardiopulmonary and renal function in thermally injured sheep. Surg Gynecol Obstet.

[20] Poindexter BJ, Pereira-Smith OM, Smith JR (May 2002). 3-Dimensional reconstruction and localization of mortalin by deconvolution microscopy. Microsc Anal.

[21] Klein GL, Poindexter BJ, Sheikh-Hamad D, Enkhbaatar P, Traber D, Bick RJ Up-regulation of the ileal calcium sensing receptor following burn injury in sheep. J. Bone Mineral Res.

